# Label-free Electrochemical Detection of ATP Based on Amino-functionalized Metal-organic Framework

**DOI:** 10.1038/s41598-017-06858-w

**Published:** 2017-07-26

**Authors:** Pengfei Shi, Yuanchao Zhang, Zhaopeng Yu, Shusheng Zhang

**Affiliations:** 1 Shandong Province Key Laboratory of Detection Technology for Tumor Makers, College of Chemistry and Chemical Engineering, Linyi University, Linyi, 276000 China; 2 Shandong Province Key Laboratory of Life-Organic Analysis, College of Chemistry and Chemical Engineering, Qufu Normal University, Qufu, 273165 China

## Abstract

A sensitive, selective and recyclable electrochemical sensor is designed for ATP detection based on amino-functionalized metal-organic framework. The functional MOF as the sensor is constructed by one-step synthesis Ce-MOF and sequentially modified on the Au electrode and conjugated with the aptamer of ATP. The presence of target ATP leads to the conformational change of aptamer strands and strong electrochemical impedance. The electrochemical sensor can detect ATP down to 5.6 nM with the linear range of 10 nm to 1000 μM. The present study is the first report on the use of MOF as an electrochemical sensor for ATP at nM level. This strategy has been successfully applied in detection of ATP in serum of cancer patients, which reveals its potential application in clinical diagnosis.

## Introduction

Adenosine triphosphate (ATP), the primary energy currency, dominates the cellular energy state and regulates cellular metabolism, which can be as an indicator for cell injury, tumors and many diseases^1, 2^. Therefore, sensitive and specific detection of ATP is extremely urgent^3^. To date, various detection methods for ATP have been designed^4–6^. Noteworthily, electrochemical biological sensing, as one of the main detection technologies, is favored due to its simple equipment, good selectivity, fast response, low cost and broad applications^7–12^. Great research efforts have been invested to find surface modification electrode in order to improve the response performance and specific interaction^13, 14^. During the reported electrochemical sensors for ATP, electrode modification is mostly based on grapheme oxide (GO), quantum dots (QDs), Au, and other nanoparticles with high surface areas and/or catalytic activity^15–19^. However, electrochemical biosensors remain suffer from lack of effective surface modification, which could not detect the desired targets with high sensitivity and unique identification^13, 20^. Thus, it is essential to explore new type of electrochemical biosensors for ATP.

Metal-organic framework (MOF), a promising class of multifunctional materials, has always been received extensive attention since 1999^21^. Due to its tunable structures, large surface areas, high thermal and chemical stabilities, MOF displays potential applications in gas storage/separation, sensors, magnetic, catalysis, drug delivery, cancer therapy, etc.^22–25^. Among them, research as biosensors based on MOF has become more and more attractive and made great progress in recent years^26^. For examples, Chen’s group utilized MOF as the sensing platform to detect ds-DNA with the detection limit 1.3 nM^27^; Kang’s group presented a novel sensing strategy based on peptide nucleic acid probes labeled with fluorophores and conjugated with an NMOF vehicle to monitor multiplexed miRNAs in living cancer cells^28^; Tang’s group developed a novel heterogeneous nano-MOF probe for H_2_S detection in HepG2 cells^29^. In contrast, MOF as electrochemical biosensors are rarely reported, which is usually caused by its low electro-activity. Yuan’s group presented a ratiometric electrochemical biosensor for sensitive detection of lipopolysaccharide by Au nanoparticles encapsulated Cu-based MOF (AuNPs/Cu-MOF)^30^. Lei’s group reported a electrochemical biosensor based on the Pt@UiO-66NH_2_, in which the Pt@UiO-66-NH_2_ was immobilized with capture DNA as a signal probe for detecting the telomerase activity in cancer cells^31^. It is not hard to find that most MOFs-based electrochemical biosensors are combined with metal NPs or post-modified functional groups, which increase the experimental complexity. Therefore, it is highly necessary to explore convenient approach to enhance the MOFs applications in the electrochemical biosensing fields.

**Figure 1:**
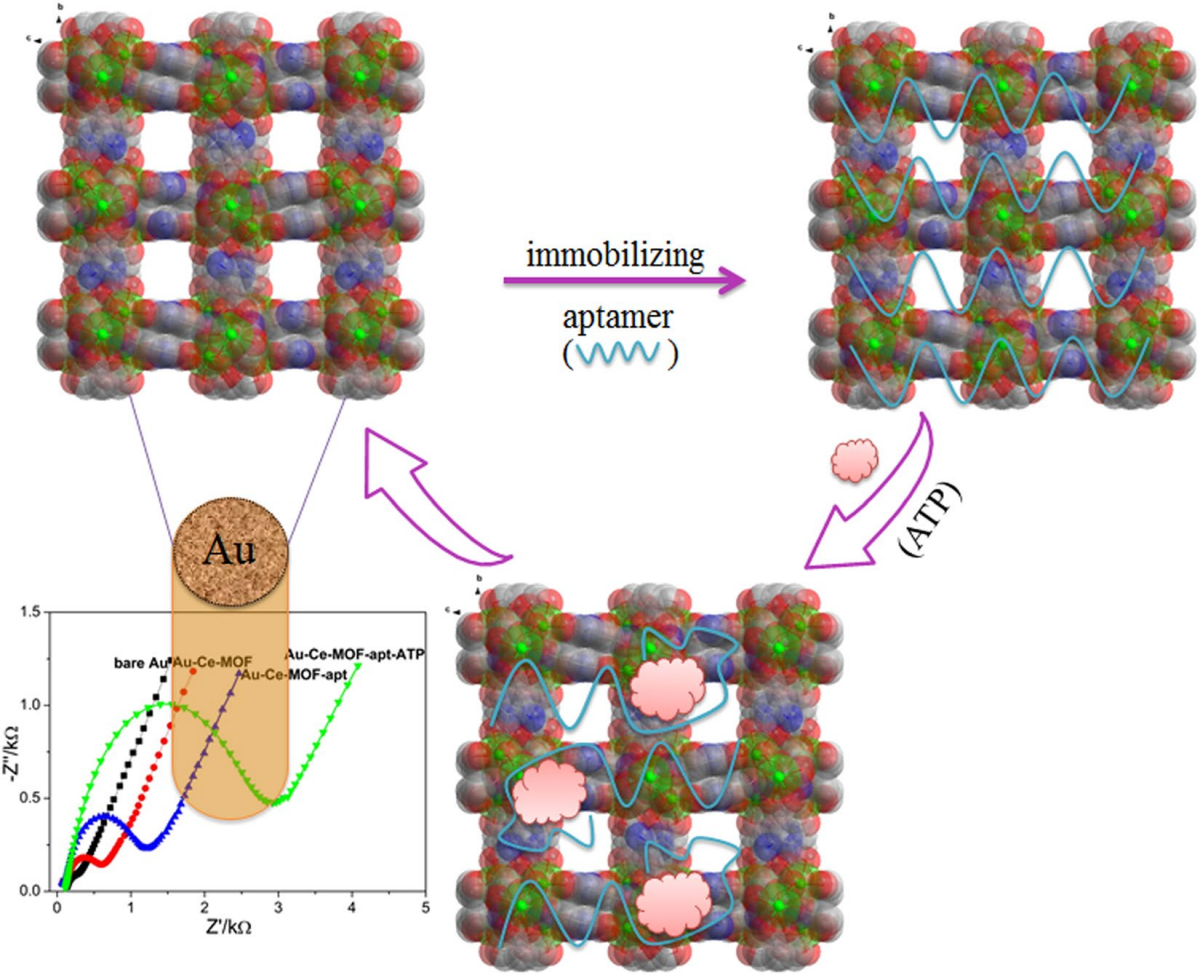
Schematic illustration of the label-free electrochemical sensor for ATP based on Ce-MOF.

In pursuit of an ideal electrochemical sensor for ATP, a 3D lanthanide MOF (Ce-MOF) was constructed from an amino-functionalized ligand (Supplementary Information, Figure S1) and Ce^3+^ ions using one-step synthesis. As shown in Fig. 1, the construction process of Ce-MOF-based electrochemical sensor for detecting ATP contains three steps: (1) the bare Au electrode was modified with Ce-MOF by weak covalent bonding^32^ and electrostatic interactions; (2) the aptamer of ATP was immobilized onto Ce-MOF *via* hydrogen bonding, π···π stacking and electrostatic interactions; (3) the specific binding affinity between ATP and its aptamer leaded to the conformational change of aptamer strands and strong electrochemical impedance in the presence of the target ATP. With the increasing concentration of the target ATP, the electrochemical impedance spectroscopy (EIS) signal increased gradually. The convenient method can detect ATP at nanomolar (nM) level. It is the first report of MOF-based electrochemical sensor for the detection of ATP. Additionally, this strategy has been successfully applied in detection of ATP in serum of cancer patients, which reveals its potential application in clinical diagnosis.

## Results and Discussion

### Characterization of the materials

**Figure 2:**
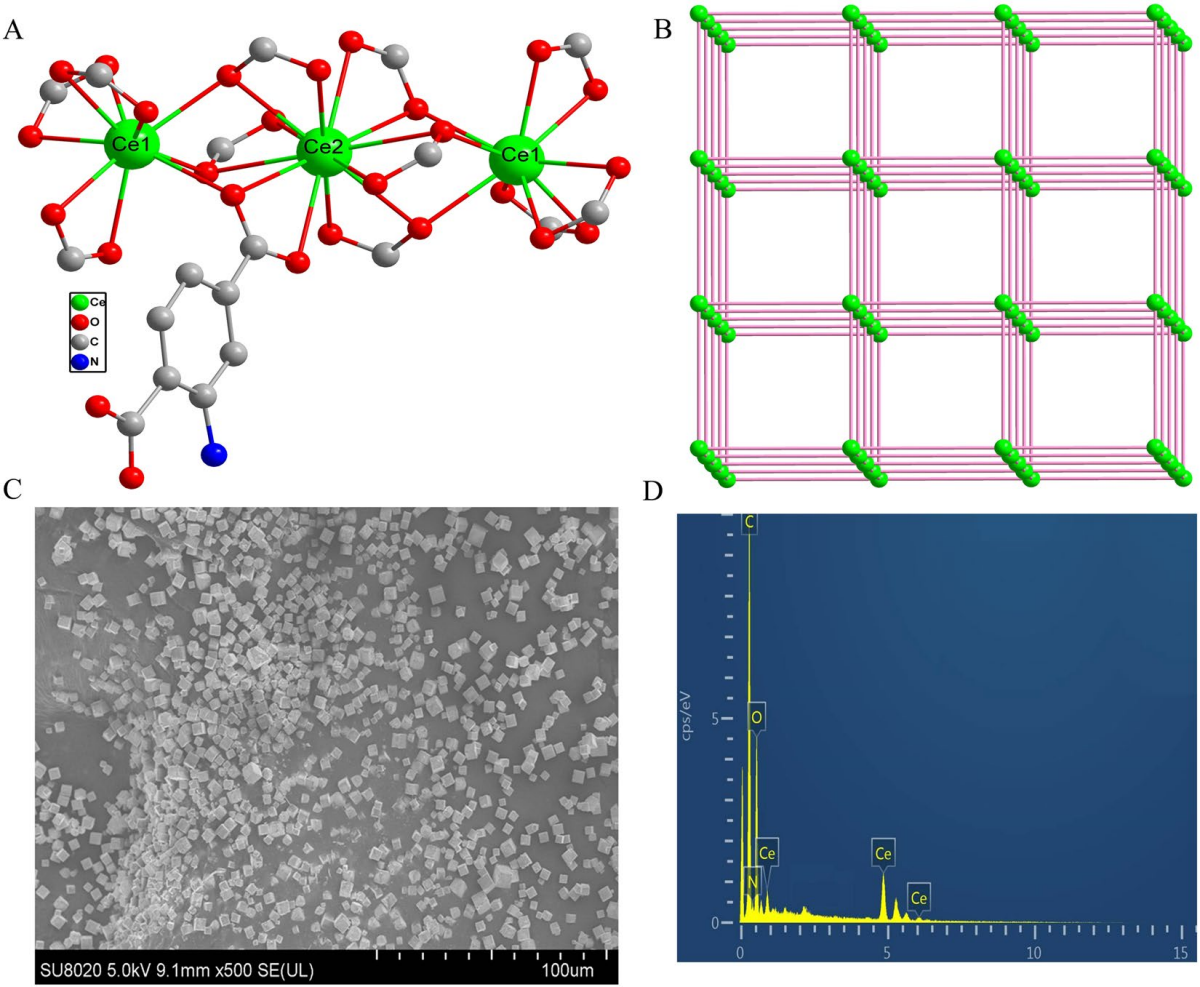
(**A**) Coordination environments of Ce^3+^ ions and H_2_L; (**B**) the 3D topological structure of Ce-MOF; (**C**) SEM image of Ce-MOF; (**D**) EDX analysis of Ce-MOF.

Single crystal X-ray diffraction analysis reveals that Ce-MOF crystalizes in cubic system with space group *Ia-3*, which is isostructural to one reported work for Nd-MOF^33^. As shown in Fig. 2A, two crystallographically independent Ce^3+^ ions are presented. Ce1^3+^ ion is nine-coordinated by nine carboxylic oxygen atoms of H_2_L. Ce2^3+^ ion is twelve-coordinated by twelve carboxylic oxygen atoms of H_2_L. Ce1^3+^ and Ce2^3+^ ions are bridged by carboxyl oxygen atoms of H_2_L into a liner trinuclear unit. The H_2_L further link the trinuclear units into a porous 3D framework. The trinuclear units are considered as nodes, which are bridged by double ligands. The Ce-MOF can be simplified to a NaCl-like topological structure (Fig. 2B). The NH_2_-groups are not coordinated, combining with the Au electrode advantageously. Scanning electron microscope (SEM) measurement shows the cubic morphology and the size of the micro-crystalline power is 3 μm (Fig. 2C). Energy-dispersive analysis of X-rays (EDX) further characterizes the element component of the Ce-MOF (Fig. 2D). The power X-ray diffraction patterns are also in conformity with the simulated pattern obtained from the single-crystal data, shown in Figure S2.

### Electrochemical biosensing

**Figure 3:**
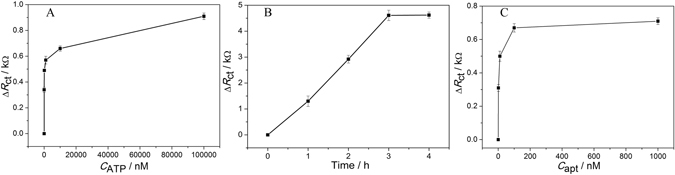
(**A**) The Δ*R*_ct_ value responses to different concentrations of ATP in absence of Ce-MOF; the Δ*R*_ct_ value responses to different reaction time between ATP and its aptamer (**B**) and aptamer concentrations (**C**) based on Ce-MOF.

EIS is one of the most generally used electrochemical techniques to monitor the electrode surface properties, which is analyzed using Zview2 software. The Randles equivalent circuit is consist of solution resistance (*R*_s_), charge-transfer resistance (*R*_ct_), constant-phase element (CPE) and warburg impedance (*Z*_w_)^34, 35^. *R*_s_ represents the effect of the concentration and the type of electrolyte solution on the ionic conductivity. CPE is a general frequency-dependent element, which is often associated with non-ideal capacitive behavior resulting from electrode roughness and inhomogeneous conductivity. *Z*_w_ depends both on the rate of diffusion in the electrode and on the frequency used to probe the impedance. A nonlinear least-squares method was used to fit and determine the parameters of the elements in an equivalent circuit (EIS Nyquist plots and equivalent circuit, Figure S3). *R*_ct_ can be directly determined by the semicircle diameter of the Nyquist plots. Any tiny changes on the electrodes surface will affect the charge transfer, which can be collected by the EIS spectroscopy. EIS is widely applied in biosensors for determining conformation changes or intermolecular interaction. In this concept, the electrochemical characterization and detection of ATP with the Ce-MOF-based sensor were carried out by EIS. As shown in Fig. 1, the EIS of bare Au electrode is almost a line due to the inexistence of hindering electron transmission. When the Ce-MOF was modified onto the Au electrode, the *R*_ct_ increased and it increased obviously after combined with the aptamer of ATP, ascribing the negative charge of nucleotide sequence, which impeded the [Fe(CN)_6_/Fe(CN)_6_]^3−/4−^ spread to the electrode surface through electrostatic repulsion. In the presence of the target ATP, the strong binding affinity between the ATP and its aptamer results in the increasing *R*_ct_. To confirm the function of Ce-MOF, a control experiment was performed, where no Ce-MOF was modified on the electrode. As predicted, the EIS of Au-apt and Au-apt-ATP with different concentrations of ATP hardly display obvious changes without Ce-MOF modified Au electrode (Fig. 3A). These results indicate Ce-MOF can be modified onto the Au electrode and well detection of ATP.

To investigate the detection of ATP based on the Ce-MOF, aptamer concentration and reaction time were optimized, as shown in Fig. 3B and C. The *R*_ct_ increased with the increasing concentration of aptamer, and it no longer changed until the concentration reached to 100 nM. The optimal reaction time between aptamer and ATP was 3 h.

### Sensitivity of the developed electrochemical biosensor based on Ce-MOF

**Figure 4:**
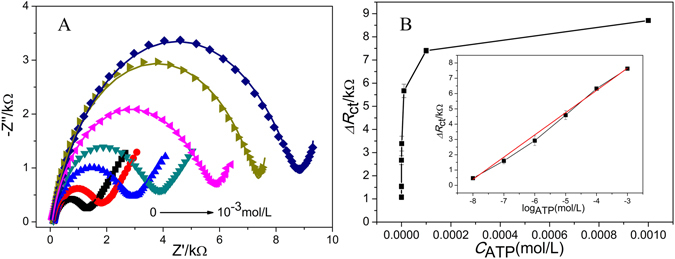
(**A**) Nyquist plots of the Au electrode modified with Ce-MOF-apt after incubation with different concentrations of ATP (0, 10^−8^ mol·L^−1^, 10^−7^ mol·L^−1^, 10^−6^ mol·L^−1^, 10^−5^ mol·L^−1^, 10^−4^ mol·L^−1^, 10^−3^ mol·L^−1^); (**B**) Dependence of Δ*R*_ct_ on ATP concentrations. Inset: Δ*R*_ct_ is linear with the logarithm of ATP concentrations ranging from 10^−8^ to 10^−3^ mol·L^−1^.

In view of the strong affinity between Ce-MOF and aptamer, detection of ATP was studied systematically. Under the optimal conditions mentioned above, detection of different concentrations ATP based on Ce-MOF are conducted and the results are depicted in Fig. 4. The *R*_ct_ values gradually increase with ATP concentrations. The increasing *R*_ct_ values may be caused by the conformation change of aptamer and the formation of the thick organic layer over the electrode surface, which block the electrons transmission of [Fe(CN)_6_/Fe(CN)_6_]^3−/4−^ to the electrode surface.

To better understand the relationship between the *R*_ct_ and concentration of ATP, a plot of Δ*R*_ct_ versus ATP concentration is summarized in Fig. 4B. The Δ*R*_ct_ represents the difference value between *R*_ct_ with different concentrations of ATP and *R*_ct_ of Ce-MOF-apt. The Δ*R*_ct_ values increase obviously with the ATP concentration increase in the range of 10^−8^~10^−3^ mol·L^−1^. The insert shows the linear part of the curve with regression equation of Δ*R*_ct_ = 1.45 log*C*_ATP_ + 12.02 (*R* ^2^ = 0.997). The linear concentration range is from 10^−8^ to 10^−3^ mol·L^−1^ and the detection limit is 5.6 nM (S/N = 3). Compared with other biosensors based on MOFs (Table 1), Ce-MOF is the first report of MOF-based electrochemical sensor for the detection of ATP, which also exhibits high sensitivity. Additionally, the analytical performance of the fabricated Ce-MOF was also compared with the other sensors for detecting ATP in previous studies (Table S1). The results show that the sensitivity of Ce-MOF for ATP is comparative to other reported methods. Importantly, quantitatively detecting ATP using the one-step synthesized Ce-MOF possesses wider linear response range than the recorded sensors for ATP.

**Table 1:** MOFs as biosensors in some reported works.

Detection method	analyte	LOD	Ref
FL	MicroRNA	10 pM	28
FL	H_2_S	16 nM	29
EC	Lipopolysaccharide	0.33 fg·mL^−1^	30
FL	H5N1 antibody	1.6 nM	36
FL	Nitric Oxide	0.03 mM	37
ECL	NT-proBNP	1.67 pg·mL^−1^	38
EC	ATP	5.6 nM	in this work

### Selectivity and recyclability of the electrochemical biosensor based on Ce-MOF in detection of ATP

**Figure 5:**
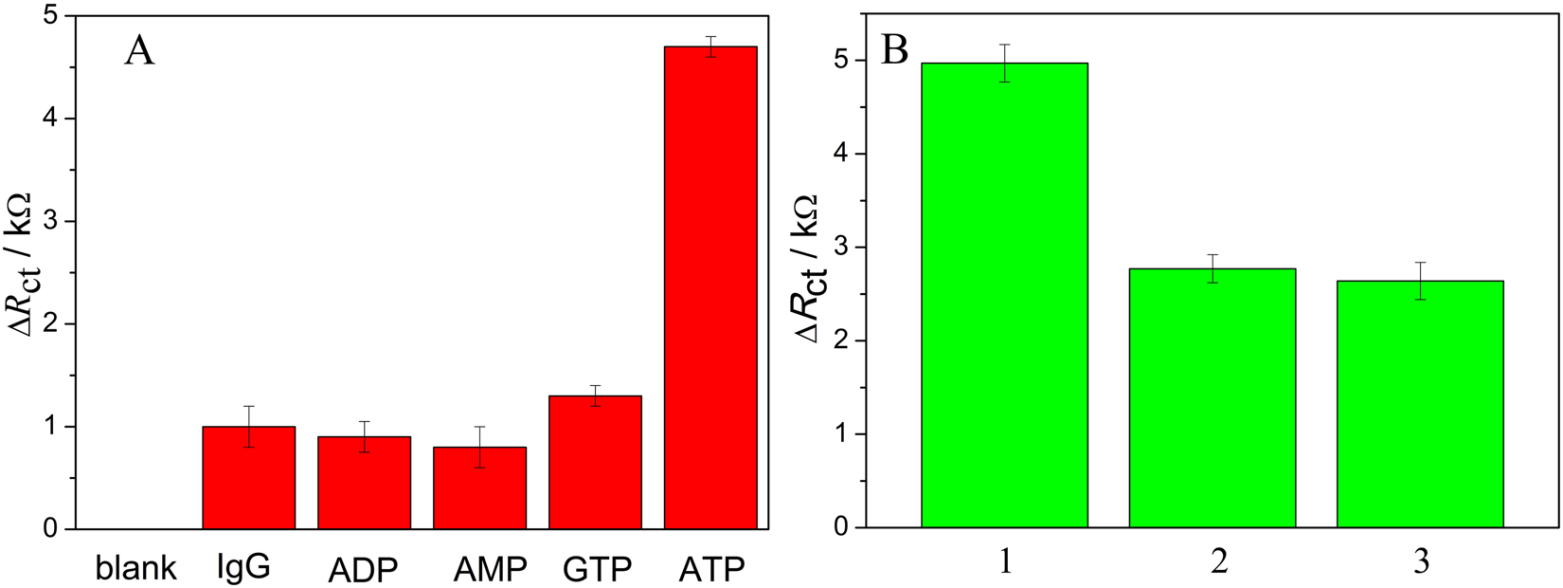
(**A**) The Δ*R*_ct_ value responses to 10^−5^ mol·L^−1^ ATP and the interferents (ATP, IgG, ADP, AMP, GTP) based on Ce-MOF-apt; (**B**) the Δ*R*_ct_ value responses to 10^−5^ mol·L^−1^ ATP for different cycles. (In the horizontal axis, **1** represents the Δ*R*_ct_ value in the presence of 10^−5^ mol·L^−1^ATP based on Ce-MOF-apt; **2** represents the Δ*R*_ct_ value in the presence of 10^−5^ mol·L^−1^ATP based on activated Ce-MOF-apt in **1**; **3** represents the Δ*R*_ct_ value in the presence of 10^−5^ mol·L^−1^ATP based on activated Ce-MOF-apt in **2**).

The selectivity and recyclability are also important performances for biosensors to accurately detect analytes in applications. The selectivity of Ce-MOF biosensor was investigated in the presence of inferences AMP, GTP, ADP and IgG. The quantitative results obtained from EIS analysis are shown in Fig. 5A. It was found that the presence of ATP resulted in an obvious increment of the resistance, although the interferents can cause a slight impedance increase, which may be explained by the non-specific adsorption between aptamer and the interferents. This result revealed that the Ce-MOF was hardly interfered with the non-specific analytes and possessed selectivity for ATP. Meanwhile, after for one detection circle, the Ce-MOF can be activated by heating. As shown in Fig. 5B, Ce-MOF can be recyclable at least two cycles. The all results indicate the biosensor Ce-MOF displays good selectivity and recyclability in detection of ATP. To evaluate the stability of Ce-MOF, the samples of after Ce-MOF sample was incubated with the aptamer and the ATP, and was activated by heating, the sample was measured by the X-ray power diffractometer. The XRD patterns are also in conformity with the simulated pattern obtained from the single-crystal data, indicating the good stability of Ce-MOF, shown in Figure S2.

### Application in analysis of samples

To assess the Ce-MOF can be used in clinic, the detection of ATP in two serum samples of cancer patients were conducted, shown in Figure S4. Accordingly to the linear equation above mentioned, the ATP levels in the two human serum samples were detected to be 4.0 μM and 6.3 μM, respectively, which was similar to the result obtained with the reported method^11^. It demonstrated that Ce-MOF could be used for quantification of ATP in clinical diagnosis.

## Conclusion

In summary, a 3D amino-functionalized MOF (Ce-MOF) was promptly constructed by one-step synthesis. It is an excellent electrochemical sensor for detecting ATP by modification onto Au electrode. The specific binding affinity between ATP and its aptamer leads to the conformational change of aptamer strands and strong electrochemical impedance in the presence of the target ATP. And the EIS signal displays a linear relation with the concentration of ATP. The convenient method can detect ATP at the nM level. It is the first report of MOF-based electrochemical sensor for the detection of ATP. Additionally, this strategy has been successfully applied in detection of ATP in human serum of cancer patients, revealing its potential application in clinical diagnosis.

## Materials and Methods

### Chemicals and reagents

2-aminoterephthalic acid (H_2_L) was purchased from J&K Ltd. (Beijing, China). CeCl_3_·6H_2_O, K_3_[Fe(CN)_6_] and K_4_[Fe(CN)_6_] were purchased from Jinanhenghua Co. Ltd. (Shandong, China). 2-propanol and bare Au electrode were purchased from Xinzheng of linyi (Shandong, China). Adenosine triphosphate (ATP), guanosine triphosphate (GTP), adenosine monophosphate (AMP), adenosine diphosphate (ADP) and human immunoglobulin G (IgG) were obtained from Sangon Biological Engineering Technology & Services Co. Ltd. (Shanghai, China). Deionized water was obtained from a Millipore water purification system (≥18 MΩ). Human serum samples were obtained from Linyi Tumor Hospital (Shandong, China). Aptamer of ATP was synthesized by Sangon Inc. (Shanghai, China) with the sequence as follows:

5′-ACCTGGGGGAGTATTGCGGAGGAAGGT-3′.

### Apparatus and Characterization

#### Apparatus

Crystallographic data of Ce-MOF was collected on a SuperNova Single Crystal Diffractometer equipped with graphite-monochromatic MoKα radiation (λ = 0.71073 Å). The data integration and empirical absorption corrections was carried out by SAINT programs. The structure was solved by direct methods. All the non-hydrogen atoms were refined anisotropically on *F* ^2^ by full-matrix least-squares techniques. Scanning electron microscopy (SEM) image was taken on an S-7800f scanning electron microscope (Hitachi, Japan). Powder X-ray diffraction (PXRD) experiment was recorded on a D/Max-2500 X-ray diffractometer using Cu-Kα radiation (λ = 1.5418 Å). Electrochemical impedance spectroscopy (EIS) spectra were measured by CHI 660E Electrochemical Workstation (Shanghai Chenhua Instrument Co., Ltd. China)

#### Preparation of MOF

The synthetic procedure is based on literature method for Nd-MOF synthesis.

Synthesis the crystal: {[Ce_3_(L)_3_(HL)_3_]·9H_2_O}_n_: NaOH (0.1 M) was added dropwise to 10 mL water solution of H_2_L (0.025 g, 0.10 mmol) adjusting the pH value to 5~6. A solution of CeCl_3_·6H_2_O (0.044 g, 0.10 mmol) in 2-propanol (10 mL) was allowed to slowly diffuse into the above solution of H_2_L through a buffering layer of 2-propanol and water (1:1). Crystals suitable for X-ray crystal analysis was obtained after three days, which are stable in air and insoluble in water and common organic solvents. Yield: 25%.

Synthesis the micro-crystalline power: NaOH (0.1 M) was added dropwise to 10 mL water solution of H_2_L (0.050 g, 0.20 mmol) adjusting the pH value to 5 ~ 6, following added CeCl_3_·6H_2_O (0.044 g, 0.10 mmol). Precipitations immediately occurred. The pale yellow precipitate was filtered and dried at 60 °C for 3 h. The yield of the reaction is close to 90%.

#### X-ray Crystallography

Using Olex2, the structure was solved using Charge Flipping and refined with the XL refinement package using Least Squares minimisation. The occupancy sum of N1, N2, N3 atoms is 1. Hydrogen atoms were not localized. Therefore, the H atoms are omitted from the refinement but not from the chemical formula. Crystal and final structure refinement of single crystal Ce-MOF is listed in Table S2. CCDC: 1531100

### EIS measurements

#### Construction of Ce-MOF-based Biosensor and Electrochemical Detection

The bare Au electrode (with a diameter of 2 mm) was polished with a slurry of Al_2_O_3_ power (0.3 and 0.05 μm) on chamois leather, followed by being rinsed with deionized water and fresh prianha solution (H_2_SO_4_:H_2_O_2_ (30% (V/V)) = 3:1) for 10 min. Then, the electrode was ultrasonic in deionized water, and dried in nitrogen atmosphere. The electrolyte solution ([Fe(CN)_6_/Fe(CN)_6_]^3−/4−^ solution, 5.0 mM) was prepared immediately before use by dissolving 1.65 g of K_3_[Fe(CN)_6_], 2.11 g of K_4_[Fe(CN)_6_], 8 g of NaCl, and 0.2 g of KCl in 1 L of deionized water. All solutions were prepared immediately prior to the experiments and stored at 4 °C. Afterwards, the electrode was washed in 0.5 M H_2_SO_4_ for 20 cyclic voltammetric circles in the range from −0.2–1.6 V. Then, the electrode was rinsed with deionized water and dried in nitrogen atmosphere again. Various concentrations of aptamer solutions were prepared using deionized water.

The construction of the Ce-MOF-based biosensor contains four steps. (1) Ce-MOF was grinded into powder and dispersed into deionized water with a concentration of 1 mg·mL^−1^, following by ultrasonic agitated for 15 min; (2) 10 μL homogenerous dispersion of Ce-MOF was dropped to the surface of bare Au electrode at 37 °C overnight; (3) The Ce-MOF-modified Au electrode was incubated in 100 nM aptamer at 37 °C for 3 h; (4) The electrode was gently washed with deionized water to remove uncombined ATP.

After that, 10 uL ATP with different concentrations was dropped to the surface of Au electrode modified with Ce-MOF-based biosensor to react for 3 h at 37 °C. Then, the electrodes were gently washed with deionized water and dried in nitrogen atmosphere.

The electrochemical workstation contains three electrodes: the counter electrode (a platinum wire), the reference electrode (Ag/AgCl with saturated KCl) and the working electrode (Ce-MOF-based biosensor modified Au electrode). All of the EIS measurements (bare Au, Au-Ce-MOF, Au-Ce-MOF-apt (aptamer), Au-Ce-MOF-apt-ATP) were carried out in 5.0 mM Fe(CN)_6_^3−/4−^ solution using the frequency range of 0.1 Hz−1 MHz with 25 mV amplitude under the open circuit potential by the Electrochemical Workstation. All of the electrochemical experiments were conducted at least in triplicate with different electrodes.

#### Selectivity of the electrochemical biosensor based on Ce-MOF

The selectivity of the biosensor based on Ce-MOF for detection of 10^−5^ mol·L^−1^ ATP was investigated in the presence of ADP, AMP, GTP, and IgG (10^−5^ mol·L^−1^ in deionized water). The detection processes were similar to that described above.

#### Detection of ATP in human serum

To investigate the feasibility of the biosensor based on Ce-MOF for clinical applications, human serum samples of two patients were tested. The human plasma was centrifuged at 5000 rpm for 10 min. The human serum samples were diluted 5-fold with deionized water before detection.

## Electronic supplementary material

Supplementary information.
