# Supplementary material for: Label-free Electrochemical Detection of ATP Based on Amino-functionalized Metal-organic Framework
Source: Sci Rep. 2017 Jul 26;7:6500. doi: 10.1038/s41598-017-06858-w (PMC5529508; doi:10.1038/s41598-017-06858-w)
Supplement: Supplementary file 1 — Supplementary information. [file 41598_2017_6858_MOESM1_ESM.doc]

**Supplementary Information**

**Label-free Electrochemical Detection of ATP Based on Amino-functionalized Metal-organic Framework**

Pengfei Shi,^1^ Yuanchao Zhang,^2^ Zhaopeng Yu,^1^ Shusheng Zhang^1,*^

^1^ Shandong Province Key Laboratory of Detection Technology for Tumor Makers, College of Chemistry and Chemical Engineering, Linyi University, Linyi 276000, China.

^2^ *School of Chemistry and Chemical Engineering, Qufu Normal University, Qufu, 273165, China.*

Pengfei Shi (E-mail: [shipengfei913@163.com](mailto:shipengfei913@163.com))

Yuanchao Zhang (E-mail: yczhang666@163.com)

Zhaopeng Yu (E-mail: yuzp0219@163.com)

Shusheng Zhang* (E-mail: [shushzhang@126.com](mailto:shushzhang@126.com))

*Corresponding author. Tel: + 86 539 8766107; fax: + 86 539 8766107.

E-mail address: shushzhang@126.com (Shusheng Zhang).

**Figure S1**. The structure of H_2_L

**Figure S2**. Power X-ray diffraction patterns

**Figure S3**. EIS Nyquist plots and equivalent circuit

**Figure S4**. Nyquist plots of the Au electrode modified with Ce-MOF-apt in two human serum samples

**Table S1**. Various detection methods for ATP in some reported works

**Table S2**. Crystal data and structure refinement for Ce-MOF


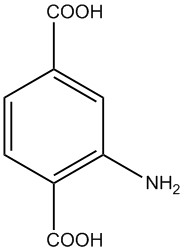


**Figure S1**. The structure of H_2_L


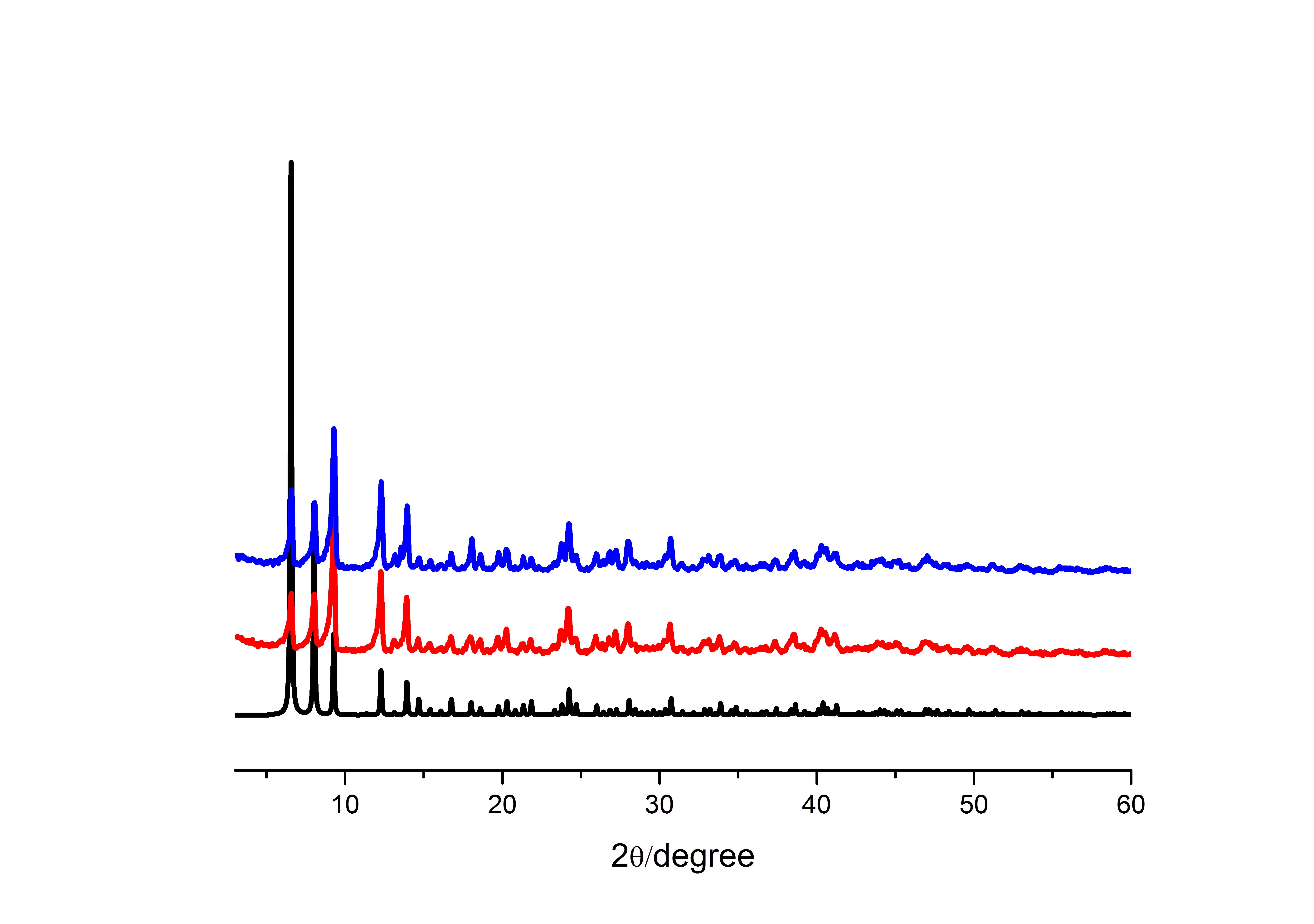


**Figure S2**. Power X-ray diffraction patterns of the samples of Ce-MOF (red line) and Ce-MOF after detection of ATP (blue line), and the simulated pattern obtained from the single-crystal data (black line).


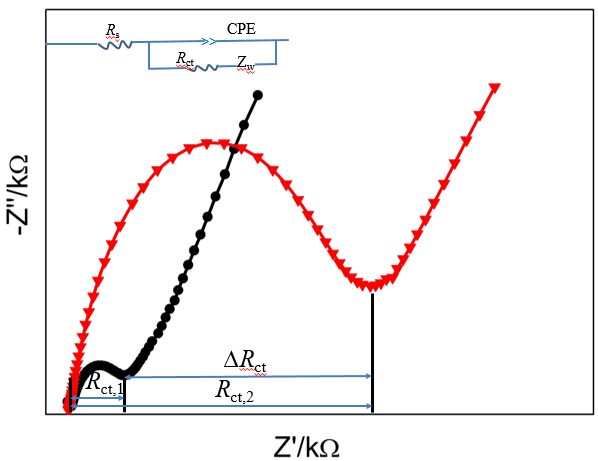


**Figure S3**. EIS Nyquist plots and equivalent circuit.


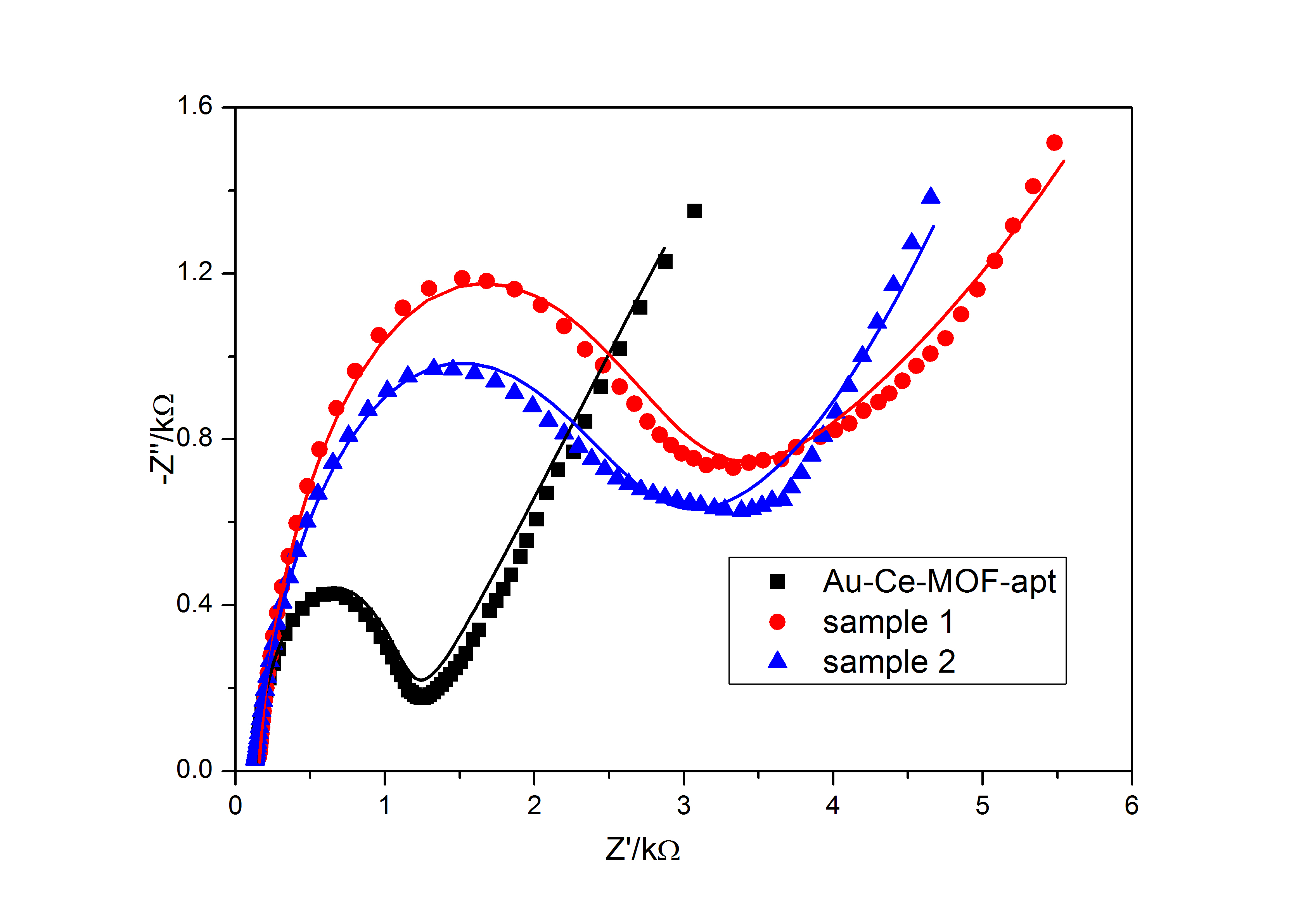


**Figure S4**. Nyquist plots of the Au electrode modified with Ce-MOF-apt in two human serum samples.

**Table S1**. Various detection methods for ATP in some reported works

| Detection method | Detection range | LOD | Ref |
| --- | --- | --- | --- |
| LSPR/gold nanorod | 10 pM-10 μM | 10 pM | 2 |
| ECL/QDs | 8-2000 nM | 7.6 nM | 3 |
| EC/exonuclease | 0.1-20 nM | 34 pM | 4 |
| EC/GO | 0.1-500 nM | 29.1 pM | 5 |
| FL/GO | 0.5-250 μM | 100 nM | 6 |
| CD/ aptasensor | 1.5-4.2 mM | 0.2 mM | 7 |
| RLS/aptasensor | 2.5-75 nM | 0.046 nM | 8 |
| EC/MOF | 10 nm-1000 μM | 3.9 nM | in this work |

**Table S2**. Crystal data and structure refinement for Ce-MOF

| Complex | **Ce-MOF** |
| --- | --- |
| Formula | C_48_Ce_3_H_48_N_6_O_33_ |
| Fw | 1508.15 |
| Crystal system | cubic |
| Space group | *Ia-3* |
| a, Å | 26.9532(7) |
| b, Å | 26.9532(7) |
| c, Å | 26.9532(7) |
| α, deg | 90.00 |
| ß, deg | 90 |
| γ , deg | 90.00 |
| V, Å^3^ | 19580.8(8) |
| Z | 8 |
| Dc, g/cm^3^ | 1.091 |
| F(000) | 6142 |
| GOF on *F*^2^ | 1.069 |
| R_1_/wR_2_(I>2σ(I)) | 0.0680/0.1641 |

References

1. Chen, X. Y. *et al*. Microporous Metal-Organic Frameworks Built on a Ln_3_ Cluster as a Six-Connecting Node. *Chem. Mater*. **17**, 2866-2874 (2005)

2. Park, J. H. High-sensitivity detection of ATP using a localized surface plasmon resonance (LSPR) sensor and split aptamers*. Biosens. Bioelectron*. **73**, 26-31 (2015).

3. Liu, Y. T. *et al*. “Off-On” Electrochemiluminescence System for Sensitive Detection of ATP via Target-Induced Structure Switching. *Anal. Chem*. **86**, 8735-8741 (2014).

4. Bao, T. *et al*. A sensitive electrochemical aptasensor for ATP detection based on exonuclease III-assisted signal amplification strategy. *Anal. Chim. Acta*. **862**, 64-69 (2015).

5. Chen, J. R. *et al*. Probe-label-free electrochemical aptasensor based on methylene blue-anchored graphene oxide amplification. *J. Mater. Chem. B*, **1**, 861-864 (2013).

6. Liu, J. H. *et al*. Graphene Signal Amplification for Sensitive and Real-Time Fluorescence Anisotropy Detection of Small Molecules. *Anal. Chem*. **85**, 1424-1430 (2013).

7. Fu, P. *et al*. A self-assembled chiral-aptasensor for ATP activity detection. *Nanoscale*, 2016, **8**, 15008-15015.

8. Chen, F. C. *et al*. Click on the bidirectional switch: the aptasensor for simultaneous detection of lysozyme and ATP with high sensitivity and high selectivity. *Sci. Rep*. **6**, 18814 (2016).
